# Increase in searches for erectile dysfunction during winter: seasonal variation evidence from Google Trends in the United States

**DOI:** 10.1038/s41443-020-00397-1

**Published:** 2021-02-11

**Authors:** Belén Mora Garijo, Jonathan E. Katz, Aubrey Greer, Mia Gonzalgo, Alejandro García López, Leslie Deane, Ranjith Ramasamy

**Affiliations:** Department of Urology, University of Miami Miller School of Medicine, Miami, FL USA

**Keywords:** Risk factors, Sexual dysfunction, Cardiovascular diseases

## Abstract

Several diseases associated with erectile dysfunction (ED), such as type 2 diabetes mellitus (T2DM) and coronary artery disease (CAD), are known to have seasonal variation, with increased incidence during winter months. However, no literature exists on whether this chronological-seasonal evolution is also present within ED symptomatology. We hypothesized ED would follow the seasonal pattern of its lifestyle-influenced comorbid conditions and exhibit increased incidence during winter months. In order to investigate the seasonal variation of ED in the United States between 2009 and 2019, Internet search query data were obtained using Google Trends. Normalized search volume was determined during the winter and summer seasons for ED, other diseases known to be significantly associated with ED (T2DM and CAD), kidney stones (positive control), and prostate cancer (negative control). There were significantly more internet search queries for ED during the winter than during the summer (*p* = 0.001). CAD and T2DM also had significantly increased search volume during winter months compared to summer months (*p* < 0.001 and *p* = 0.011, respectively). By contrast, searches for kidney stones were significantly increased in the summer than in the winter (*p* < 0.001). There was no significant seasonal variation in the relative search frequency for prostate cancer (*p* = 0.75). In conclusion, Google Trends internet search data across a ten-year period in the United States suggested a seasonal variation in ED, which implies an increase in ED during winter. This novel finding in ED epidemiology may help increase awareness of ED’s associated lifestyle risk factors, which may facilitate early medical evaluation and treatment for those at risk of both ED and cardiovascular disease.

## Introduction

Erectile dysfunction (ED) is defined as the recurrent inability to attain or maintain an erection sufficient for satisfactory sexual activity [1]. ED is a common worldwide clinical problem affecting approximately 30 million men in the United States [2]. Several diseases including type 2 diabetes mellitus (T2DM) and coronary artery disease (CAD) have been shown to share pathophysiological risk factors and be significantly associated with ED [3]. Interestingly, both T2DM [4] and CAD [5] are known to have seasonal variation, with increased incidence during winter months. Lifestyle changes during the winter may underlie this phenomenon, as it has been well characterized that individuals gain weight over the holiday season (Thanksgiving - New Year’s) [6]. This could be expected to negatively impact the aforementioned conditions. To our knowledge, the seasonal variation or chronological-seasonal evolution of ED has yet to be explored. Gaining knowledge of the seasonal variation and time trends in ED may improve awareness of risk factors and surveillance methods, in addition to facilitating prevention and management strategies.

With 80% of all American adults conducting online searches for health information on health conditions prior to seeking physician consultation [7], the popularity of the internet and search engines has opened a new avenue for epidemiological research. Previous studies have used internet search trends data, specifically Google Trends, a free publicly available tool for analyzing search query data, to investigate temporal patterns of several health conditions, including, T2DM [8], CAD [9], influenza [10], depression [11], restless leg syndrome [12], and kidney stones [13].

The purpose of our investigation was to utilize internet search query data from the United States over a 10-year period to test the hypothesis that there is seasonal variability to ED. Given that diseases such as CAD and T2DM, which ED is frequently associated with and have a well-established predisposition for winter months, we hypothesized that there would be a seasonal pattern to ED, with relative increased frequency during winter months. Additionally, we tested kidney stones and prostate cancer (PCa) as a positive and negative control, respectively.

## Methods

A retrospective study was designed to evaluate seasonal variation for the search topics: “erectile dysfunction”, “type 2 diabetes mellitus”, “coronary artery disease”, “kidney stones”, and “prostate cancer”. As this is publicly available data, it fulfills criteria for Institutional Review Board (IRB) exemption, and an IRB waiver was obtained.

### Search queries

To improve reproducibility of our results [14], we report the following search query details: On February 24, 2020, we queried Google Trends and downloaded the data for the following search topics: erectile dysfunction, coronary artery disease, type 2 diabetes, prostate cancer, and kidney stones. A search topic on Google Trends automatically includes results for a group of terms that share the same concept in any language (e.g., “erectile dysfunction” search topic includes results for “impotence”, “disfunción eréctil, which is “erectile dysfunction” in Spanish, etc.) [15]. No quotation marks were used for the search. For each year from 2009 to 2019, we searched within the United States from December 21st to March 20th for the winter season and from June 20th or 21st to September 22nd or 23rd for the summer season. Seasons were determined based on historic national records according to the Equinoxes and Solstices for the years 2009 to 2019 [16]. It is important to note that for the winter of any year, a part of the winter season includes the final days of the year prior (e.g., the Winter of 2010 begins at the end of 2009).

Each Google Trend data point represents the proportion of searches for the queried topic by the total searches in the USA, during the time range selected. The result is a relative search frequency on a scale ranging from 0 to 100. Each proportion can represent millions of searches occurring in the specified location and time frame [14].

### Outcomes

Our primary outcome was to compare proportion of searches for ED in the winter and summer months. Secondly, we wanted to validate and characterize the size of seasonal variation for coexistent conditions such as T2DM and CAD. Finally, we included the topics “kidney stones” and “prostate cancer” as urologic conditions with and without known seasonal variability. Our positive control, “kidney stones”, was selected as a benchmark by which to measure any seasonal variation [17]. Furthermore, PCa was included as a negative control [18], if positive it would suggest diseases with no known reason for seasonal variation may be more commonly searched for in the Winter.

### Statistical analysis plan

By means of paired sample *t*-tests, mean search activity during winter months was compared to mean search activity during summer months for a 10-year period for each of the aforementioned diseases. Data were analyzed using IBM SPSS Statistics Software 24.0.

## Results

**Fig. 1:**
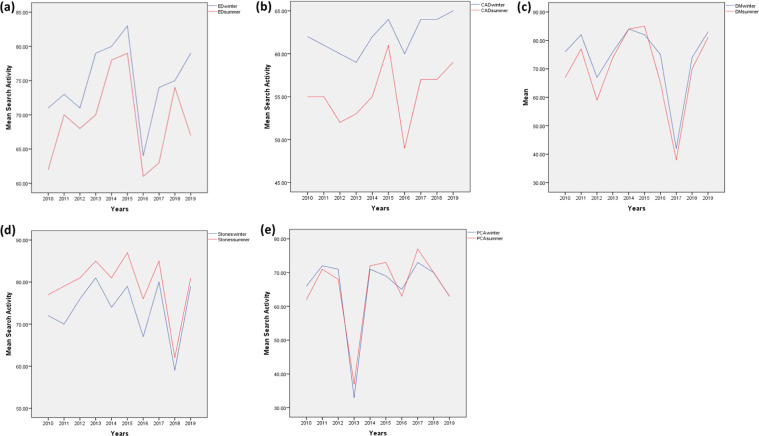
Health internet search activity trends in the United States during winter and summer months from 2009 to 2019. **a** Erectile dysfunction, **b** coronary artery disease, and **c** type 2 diabetes mellitus show significantly greater mean search activity during winter (blue) than summer (red) throughout a ten-year period. **d** Kidney stones show greater mean interest search activity during summer (red) than winter (blue) throughout a ten-year period. **e** Prostate cancer shows no difference in mean internet search activity during winter (blue) and summer (red) throughout a ten-year period.

Over the last ten years, there have been significantly more Google searches for ED during the winter than during the summer, with a 6% increase in searches in the winter compared to the summer (mean search frequency 74.9 ± 5.6 and 69.2 ± 6.3, respectively, *p* = 0.001) (Table 1, Fig. 1a). Similarly, there was a statistically significant increase in average search queries for CAD and T2DM in the winter in comparison to the summer (CAD: 62.1 ± 2.1 vs 55.3 ± 3.5, *p* < 0.001; T2DM: 74.1 ± 12.4 vs 70.0 ± 14.1, *p* = 0.011) (Table 1, Fig. 1b, c). The search frequency for kidney stones, used as a positive control, was significantly greater in the summer than in the winter, with a 6% increase in searches in the summer compared to the winter (79.4 ± 7.1 vs 73.7 ± 6.9, *p* < 0.001) (Table 1, Fig. 1d). Finally, there was no significant difference between mean relative search frequency for PCa in the winter than in the summer (65.3 ± 11.8 vs 65.6 ± 11.2, *p* = 0.75) (Table 1, Fig. 1e).

**Table 1:** Results of *t*-test & descriptive statistics for mean internet search frequency during winter and summer from 2010 to 2019 in the United States for erectile dysfunction, coronary artery disease, type 2 diabetes, prostate cancer and kidney stones.

	Paired samples test								
	Paired differences								
	Mean	Std. deviation	Std. error mean	95% CI					
				Lower	Upper	*t*	df	p	Sig. (2-tailed)
ED Winter –ED Summer^a^	5.70	4.08	1.29	2.78	8.62	4.41	9	0.0017	.002
CAD Winter – CAD Summer^b^	6.80	1.99	0.63	5.38	8. 22	10.81	9	1.86e−6	.000
T2DM Winter – T2DM Summer^c^	4.10	4.09	1.29	1.17	7.03	3.17	9	0.011	.011
PCa Winter – PCa Summer^d^	−0.30	2.94	0.93	−2.41	1.81	−0.32	9	0.75	.755
Stones Winter – Stones Summer^e^	−5.70	2.45	0.72	−7.45	−3.95	−7.35	9	4.31E−8	.000

Paired samples *t*-test results comparing difference between mean search activity for.

^a^Erectile dysfunction (ED).

^b^Coronary artery disease (CAD).

^c^Type 2 diabetes (T2DM).

^d^Prostate cancer (PCa), and

^e^Kidney stones in the summer and winter over a ten-year period in the United States.

When comparing the magnitude of the seasonal variability, using kidney stones as the standard: ED, CAD and T2DM had 0.60, 1.47, and 0.43 times the amount of search volume seasonal variability, respectively.

## Discussion

The current study demonstrates an increase in search volume activity for ED during the winter, as compared to the summer, in the United States over the past ten years. The same methodology was also successfully used to confirm previously established seasonal patterns of increased morbidity in the winter for T2DM [8] and CAD [9], decreased morbidity in the winter for kidney stones [17], and no effect on PCa [18]. To our knowledge, our study is the first to suggest a seasonal variation in the epidemiology of ED. For perspective, when comparing the magnitude of the seasonal variability among the different diseases tested, the effect size for ED was 60% of the effect size for kidney stones.

The mechanisms underlying the seasonal patterns of ED are likely due to its associated comorbidities and risk factors. Organic ED (with an underlying physical etiology) and CAD are closely linked due to their shared pathophysiologic mechanism, namely endothelial dysfunction and atherosclerosis leading to reduced blood flow [19]. In fact, ED symptoms have been shown to precede CAD symptoms by 2–3 years, and precede a cardiovascular event (myocardial infarction or stroke) by 3–5 years [19]. Research has already demonstrated that cardiovascular disease (CVD) has a well-established predilection for winter months. CVD-related hospitalizations and mortality reach maximum numbers in the winter (January), with cardiac event rates in winter being typically 10–20% greater than during summer [5]. Similarly, Ishii et al. (2001) demonstrated that the mean hemoglobin A1c level for 39 patients with Type 2 Diabetes was elevated by 0.5% in winter compared with the period between spring and autumn. The present study reinforces these previously reported seasonal patterns in CAD and T2DM using online search data.

Potential risk factors associated with worse CVD in the wintertime include, increased dietary calorie intake, decreased physical activity, and increased alcohol consumption [5, 20], which are also likely to negatively impact erectile function. Awareness of the shared seasonal variation of ED with its associated risk factors may allow the implementation of more timely prevention and management strategies for symptomatic patients.

Another plausible explanation for the seasonality of ED may relate to an increase in sexual activity during winter. A study conducted by Seiver (1985) examined 30 years of birth records in the United States and concluded that children tend to be conceived more frequently in winter months (especially December) than during any other month [21]. This increase in sexual activity during cold winter months increases the likelihood of discovering ED symptoms and boosting online search queries. Other explanations may be related to fluctuations in testosterone levels, holiday times, seasonal affective disorder, and hours spent indoors.

Despite the consistency of our findings with previously established seasonal patterns of common health conditions like CAD, T2DM, and kidney stones, our study has several limitations. Online health-information seeking behavior for particular search terms cannot guarantee the person searching is experiencing those symptoms at the time. Additionally, the current study did not distinguish between the different geographic climates that exist within the United States. A state-by-state or regional comparison may have more accurately reflected seasonal variation for each disease. Moreover, income level and demographics characteristics may influence the likelihood of using Google as a source of health information. This is particularly relevant for ED because the prevalence of this disease increases with advancing age, and seasonal trends observed in our study may under sample geriatric populations, as they may be less inclined to use Google. It is also important to note that for each disease there is a sharp decline in search volume per year over the course of 2015 to 2017 (Fig. 1). Though the annual variation may have several explanations related to the Google Trend technology or to actual variations in the US population’s usage of Google to research a specific disease, the ratio of winter to summer queries remains approximately constant. This suggests that though there is annual variation, our findings regarding seasonal variation remain robust to the impact of annual variation.

Internet search query surveillance is rapidly expanding into many areas of epidemiological research. The present study reports a significant increase in overall Google search activity for ED during the winter in the United States. However, future studies are needed to determine whether lifestyle changes in the winter underlies this phenomenon.

## Conclusions

Evidence from Google search queries across a ten-year period in the United States demonstrates a seasonal variation in ED with an increase in winter months. This phenomenon may be influenced by lifestyle changes in the winter months, which could explain why similar conditions known to be affected by lifestyle demonstrate similar seasonal variability. This novel finding in ED epidemiology may lead to increased awareness of ED’s seasonal variation and associated lifestyle risk factors, which may aid in identifying and treating those at risk of both ED and CVD.
